# Characterization of a Novel Third-Generation Anti-CD24-CAR against Ovarian Cancer

**DOI:** 10.3390/ijms20030660

**Published:** 2019-02-03

**Authors:** Rüdiger Klapdor, Shuo Wang, Michael Morgan, Thilo Dörk, Ulrich Hacker, Peter Hillemanns, Hildegard Büning, Axel Schambach

**Affiliations:** 1 Department of Gynecology and Obstetrics, Hannover Medical School, 30625 Hannover, Germany; wangshuo1022@gmail.com (S.W.); doerk.thilo@mh-hannover.de (T.D.); hillemanns.peter@mh-hannover.de (P.H.); 2 Institute for Experimental Hematology, Hannover Medical School, 30625 Hannover, Germany; morgan.michael@mh-hannover.de (M.M.); ulrich.hacker@medizin.uni-leipzig.de (U.H.); buening.hildegard@mh-hannover.de (H.B.); 3 Cluster of Excellence REBIRTH, Hannover Medical School, 30625 Hannover, Germany; 4 University Cancer Center Leipzig (UCCL), University Hospital Leipzig, 04103 Leipzig, Germany; 5 Division of Hematology/Oncology, Boston Children’s Hospital, Harvard Medical School, Boston, MA 02115, USA

**Keywords:** ovarian cancer, chimeric antigen receptor, CD24, immunotherapy, dual CAR

## Abstract

Novel therapeutic approaches against ovarian cancer (OC) are urgently needed because of its high rate of recurrence even after extensive surgery and multi-agent chemotherapy. We aimed to develop a novel anti-CD24 chimeric antigen receptor (CAR) as an immunotherapeutic approach against OC cells and cancer stem cells (CSC). CSC represents a subpopulation of the tumor characterized by enhanced chemoresistance as well as the increased capability of self-renewal and metastasis. We designed a codon-optimized third-generation CAR containing the highly active single chain variable fragment (scFv) “SWA11” against CD24. We equipped the human NK-cell line NK-92 with the anti-CD24 CAR and an anti-CD19 control CAR using lentiviral transduction. Engineered NK-92 cells showed high cytotoxic activity against CD24-positive OC cell lines (SKOV3, OVCAR3). This effect was restricted to CD24-expressing cells as shown after lentiviral transduction of CD24-negative cell lines (A2780, HEK-293T) with CD24 transmembrane proteins. Additionally, NK-92 cells equipped with our novel anti-CD24 CAR were highly effective against patient-derived primary ovarian cancer cells. The activation of NK cells was shown by specific IFNγ secretion upon antigen stimulation. To further reduce possible off-target effects in vivo, we applied a dual-CAR approach using an anti-CD24-CD28-41BB fusion protein linked via a 2A sequence to an anti-mesothelin-CD3ζ-CAR. The dual-CAR was simultaneously active against CD24 and mesothelin expressing cells. Our novel anti-CD24-CAR showed a highly cytotoxic effect against OC cell lines and primary OC cells and will be evaluated in future in vivo trials as a promising immunotherapeutic approach against OC.

## 1. Introduction

Among gynecologic malignancies, ovarian cancer (OC) has the highest mortality. Despite the use of ultrasounds, tumor markers or regular gynecologic examinations, OC is mostly detected at advanced tumor stages with tumor spread over the entire abdominal cavity. Extensive abdominal surgery followed by chemotherapy usually reduces the tumor residuals to a microscopic size. However, chemotherapy-refractory OC recurs within months to years after this treatment in most patients and leads to subsequent death [1,2]. Thus, novel therapeutic strategies are urgently needed. Current research in this direction focuses on cell- or antibody-based immunotherapeutic approaches due to the possibilities of specifically targeting tumor cells of interest. Cancer stem cells (CSC) represent a very interesting target for immunotherapy, since these cells are characterized by high resistance against chemotherapy and an increased capability of self-renewal [3,4,5]. Of relevance, frequently described CSC markers in OC are CD133, CD44, CD24, CD117, and their elevated expression, especially for CD44, CD24 or CD133, was shown to be associated with poor clinical outcome in OC patients [3]. Among these, CD24 is a very promising target, since it is rarely expressed in normal human tissue and is predominantly found in hematologic cells [6,7]. Of broader interest, CD24 is expressed in a variety of human cancers and is associated with poor survival [6,7,8]. Antibody-based treatments against CD24 have been evaluated in preclinical studies [9,10,11]. Noteworthy, SWA11 was identified as a very promising anti-CD24 antibody with high affinity, strong cytotoxic activity and low off-target effects [11,12]. Therefore, we selected the single chain variable fragment (scFv) from this antibody for our newly designed chimeric antigen receptor (CAR) against CD24.

CARs, as fusions of a scFv with the intracellular signal transduction domains of the T-cell receptor, represent a very promising strategy in adoptive immunotherapy [13,14]. After engineering immune effector cells, like T-cells or NK cells, with antigen-specific CARs, these cells acquire the ability to detect and specifically kill antigen-presenting cells. Recently developed CARs against several OC-antigens showed promising results in vitro and in vivo [15,16,17]. Mesothelin is frequently expressed in OC and other epithelial cancers [15]. However, these first-generation CARs often lacked functionality in clinical trials. Second and third-generation CARs were developed, which differ from first generation CARs in that they contain one or two costimulatory domains such as those derived from CD28 and/or 4-1BB in addition to the CD3ζ signaling domain, leading to improved survival and proliferation of the effector cells.

In this project, we developed a novel CD24 specific third-generation CAR, which we characterized and analyzed for its functionality in OC cell lines and primary patient-derived OC cells.

## 2. Results

### 2.1. Generation of a New Third-Generation Anti-CD24 CAR

**Figure 1:**
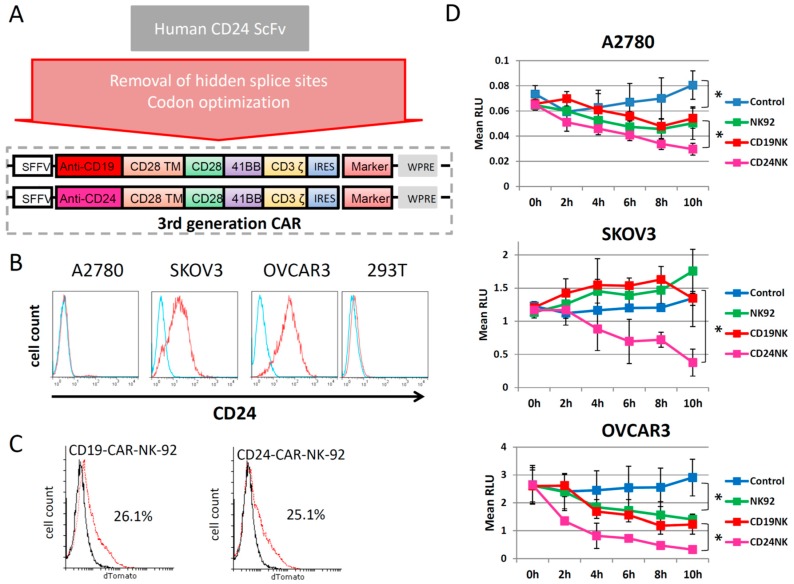
A new codon-optimized anti-CD24-CAR kills CD24 expressing ovarian cancer cells. (**A**) Schematic illustration of the structure of the tested CAR. (**B**) FACS analysis of the antigen expression of CD24 in different ovarian cancer cell lines. (**C**) FACS analysis of NK-92 cells engineered with their respective CAR (dTomato). (**D**) Fluoroskan results showing the killing effect of the engineered NK cells in ovarian cancer cell lines. In short, GFP-positive ovarian cancer cells were seeded in 96-well plates at previously determined densities (A2780, 2 × 10^4^ cells/well; SKOV3 and OVCAR3 1.5 × 10^4^ cells/well). NK-92 and CAR-NK-92 cells were added at the effector/target (E/T) ratio 5:1 one day later. Cells were lysed with SDS, and fluorescence intensity was measured at excitation 485 nm/emission 520 nm using Fluoroskan Ascent™ FL. RLU abbreviates relative light units. Values represent the mean from two separate experiments each containing three samples. * indicate *p*-values < 0.05.

The scFv of the monoclonal antibody SWA11, which is specific for the LAP epitope of the protein core of human CD24, was selected for our anti-CD24 CAR [12]. After codon optimization and removal of cryptic splice sites, the anti-CD24 scFv was cloned into a backbone of an already established third-generation CAR, containing codon-optimized CD28, 4IBB and CD3ζ CAR transmembrane, co-stimulatory and signal transduction domains (Figure 1A). The CAR was inserted into a lentiviral 3rd generation SIN vector backbone. As a marker, we used dTomato, a dimeric variant of a red fluorescent protein, which was co-expressed with the CAR sequence via an IRES sequence (Figure 1). After production of the lentiviral vector, which was pseudotyped with RD114/TR, we transduced NK-92 cells. We used A2780, SKOV3 and OVCAR3 ovarian cancer cell lines as target cells. Flow cytometric quantification of CD24 expression levels in the different cell lines showed relatively high expression in SKOV3 and OVCAR3 cells, while A2780 and 293T cells lacked CD24 expression (Figure 1B). In the following experiments, we analyzed the killing activity of CAR-NK-92 cells against the different cell lines in vitro.

### 2.2. The novel CD24-CAR-NK-92 Cells Show Specific Killing of Ovarian Cancer Cell Lines

FACS analysis showed comparable proportions of transduced cells for CD19-CAR and CD24-CAR-NK-92 cells, as shown in Figure 1C. After marking the selected cell lines with GFP by lentiviral transduction, we performed a co-incubation with CAR-engineered NK-92 cells (Figure 1D). We selected an E/T ratio of 5:1 and observed killing ability as the percent survival over 10 h with the Fluoroskan reader. A2780 cells, which were mostly negative for CD24 (6% positive cells), served as control cells. There was a significant difference in viability of A2780 cells cultured alone as compared to those co-cultivated with untransduced NK-92 control cells (*p* < 0.001). However, no differences in A2780 survival were observed between those co-cultured with CD19 CAR NK cells or untransduced NK cells (*p* = 0.587). Interestingly, co-incubation of A2780 cells with CD24-CAR-NK-92 cells resulted in slightly, but significantly, enhanced killing when compared to co-culture with untransduced and CD19-CAR control NK cells (*p* < 0.001). In contrast, co-incubation of SKOV3 and OVCAR3 cells with CD24-CAR-NK-92 cells resulted in specific OC cell killing, which clearly outperformed the anti-OC effects caused by the unmodified NK-92 control cells (*p* < 0.01). Remarkably, the CD24-CAR-NK-92 cells completely eradicated SKOV3 and OVCAR3 tumor cells, which highly express CD24. These results were confirmed by fluorescence microscopy (data not shown).

### 2.3. Specific Killing of Engineered NK Cells

**Figure 2:**
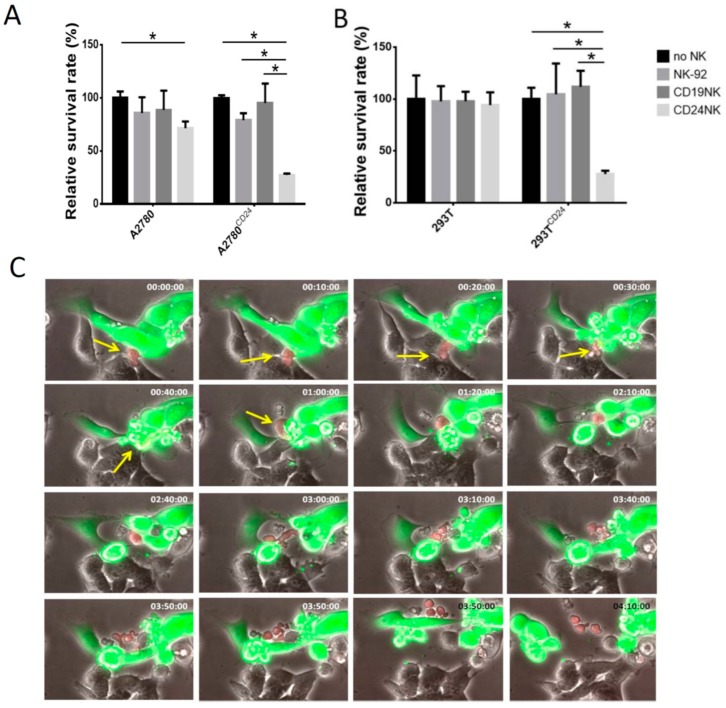
Cytotoxic activity of engineered anti-CD24-CAR-NK-92 cells is restricted to antigen-expressing cells (**A**,**B**). After lentiviral transduction of A2780 (6.6% CD24 positive) (**A**) and HEK-293T cells (CD24 negative) (**B**), cells were seeded in 96-well plates and co-incubated with the indicated NK cells at an E/T ratio of 5:1. The graphics illustrate the Fluoroskan results after 24 h incubation. * indicate *p* < 0.05 (unpaired *t*-test). Values represent the mean from two separate experiments, each containing three samples. (**C)** Serial photos of a co-culture of CD24 positive OVCAR3 cells (green) with CD24 negative HEK-293T cells (grey) under the fluorescence microscope. Target cells (OVCAR3 and HEK-293T) were mixed at a ratio of 1:1. Anti-CD24-CAR-NK-92 cells were then added with an E/T ratio of 1:1. Anti-CD24 NK-92 cells (red) specifically kill only CD24 positive OVCAR3 cells and later proliferate. Arrows point to engineered NK-92 cells. Time stamp indicates hh:mm:ss.

Due to the high killing efficiency of CD24-specific NK cells against SKOV3 and OVCAR3 cells, we performed the following experiments to show the specificity of the killing effect of CD24-CAR-NK-92 cells in cancer cells. Therefore, we equipped CD24-negative cell lines (A2780, HEK-293T) with CD24 transmembrane proteins by lentiviral transduction, in which GFP served as a marker for transduction. Again, we analyzed killing effects with Fluoroskan. Figure 2A,B show that our newly designed anti-CD24-CAR endows NK-92 cells with the ability to specifically kill only antigen-presenting cells. Similar to the previous experiment, we observed a slight killing effect in native A2780 cells, which express CD24 in a small proportion of cells (*p* < 0.01, compared to control cells). To investigate the selectivity of engineered NK cells and kinetics of target cell killing in more detail, we mixed antigen-expressing cells (OVCAR3) with HEK-293T as control cells that do not express CD24. The co-culture was observed using live cell imaging, with fluorescent and phase-contrast images taken every 10 min (Figure 2C, videos in Supplementary Materials). The evaluation of serial images of one microscopic field showed that CD24-negative HEK-293T remained unaffected by CD24-specific NK cells and continued to grow. In contrast, CD24-positive OVCAR3 cells (green) were rapidly lysed by engineered NK cells. Interestingly, we were also able to observe the expansion of the engineered NK cells after killing of cancer cells (Figure 2C, lines 3 and 4).

### 2.4. Engineered NK Cells Specifically Kill Primary Patient-Derived Ovarian Cancer Cells

**Figure 3:**
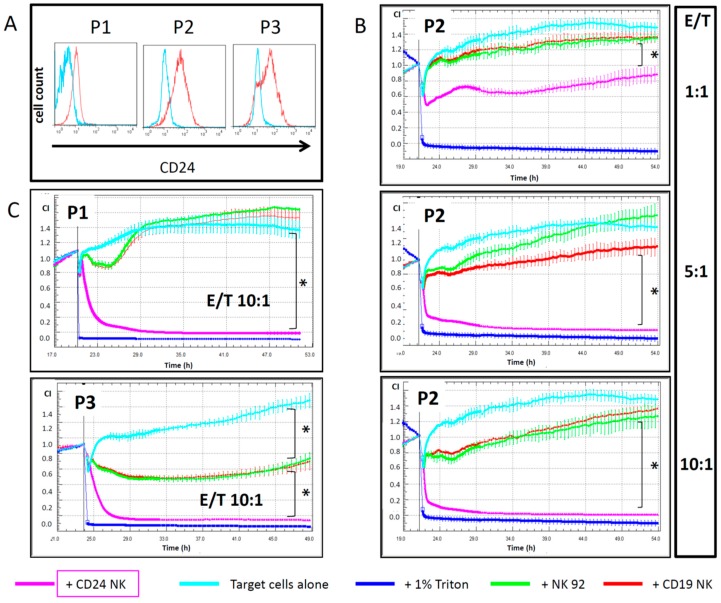
Anti-CD24-CAR-NK-92 cells exhibit strong killing activity against primary OC cells. (**A**) Flow cytometric quantification of CD24 expression in three different primary ovarian cancer cell samples. These cells were harvested from consecutive ascites samples from one patient before (P1) or during chemotherapy (P2 and P3). (**B**) Cytotoxic effects of engineered NK-92 cells in primary ovarian cancer cells (P2) as measured by xCELLigence. Per well, 1 × 10^4^ primary OC cells were seeded. E/T indicates the specific effector/target cell ratios. (**C**) xCELLigence results for P1 and P3 cells at an E/T ratio of 10:1. Values represent the mean from two separate experiments, each containing three samples. * indicate *p*-values < 0.01 for the 10 h time point (ANOVA).

We cultured OC cells directly from ascites of an OC patient collected at three different time points during treatment (P1-3) and analyzed the CD24 expression pattern at each time point (Figure 3A). P1 was collected before Paclitaxel treatment; P2 and P3 were harvested 20 and 30 days after initiation of Paclitaxel treatment, respectively. The primary OC cells showed a high expression of CD24, which even increased over time and after chemotherapy cycles. Since these cells were not marked for fluorescence analysis, we used the xCelligence real-time impedance analyzer to assess the killing effect mediated by engineered NK cells. We co-incubated primary OC cells with control and CAR-engineered NK cells over 24 h and analyzed different effector-/target(E/T)-cell ratios as indicated in Figure 3B,C. CD24-CAR-NK-92 cells showed strong killing activity against primary OC cells, with a rapid and strong killing effect even at an E/T ratio of 1:1. Anti-OC activity increased with higher E/T-ratios, with almost complete elimination of primary OC cells at E/T ratios of 5:1 and 10:1. Even after 2 h of incubation, specific CD24-CAR-NK-92 cells led to a significantly increased killing of target cells compared to control CD19-CAR-NK-92 cells (*p* < 0.01). Interestingly, the NK-92-mediated killing effect, including the unspecific killing effect of unmodified control NK-92 cells, CD19-CAR-NK-92 cells and CD24-CAR-NK-92 cells, was stronger in primary OC cell samples P2 and P3 as compared to sample P1, and thus correlated with CD24 expression levels in OC patient samples.

### 2.5. Activation of NK Cells

**Figure 4:**
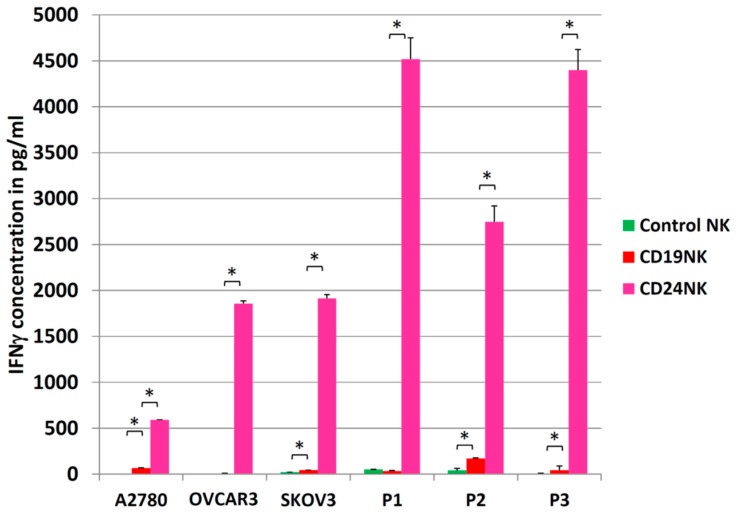
Anti-CD24-CAR-NK-92 cells produce high levels of IFNγ upon co-incubation with CD24-expressing cancer cells. IFNγ concentration in cell-free supernatants after co-culture for 24 h with an E/T ratio of 5:1 was measured by ELISA. Values represent the mean from two separate experiments each containing three samples. Control NK are unmodified NK-92 cells, CD19NK are CD19-CAR-NK-92 cells, and CD24NK are CD24-CAR-NK-92 cells. * indicate *p*-values < 0.01 (independent *t*-test).

Activation of NK cells was quantified by measuring IFNγ secretion levels. Engineered NK cells and control cells were co-cultured with target cells at an E/T ratio of 5:1 for 24 h. After co-incubation, we collected cell-free supernatants and performed an IFNγ-specific ELISA (Figure 4). CD24-CAR-NK-92 cells specifically produced high amounts of IFNγ upon target presentation by cancer cells. Even in very low expressing A2780 cells (6.6% of cells express CD24), an eight-fold increase in IFNγ expression was detected for CD24-CAR-NK-92 cells compared to control NK cells (*p* < 0.05). In P1 and P3 cells, we detected a 136- and 102-fold increase in IFNγ expression with CD24-CAR-NK-92 cells, respectively. Background activation of unmodified control NK-92 cells was highest against primary ovarian cancer cells (P2) leading to only a 16-fold increase of IFNγ expression. Interestingly, the CD24-specific NK cells also showed the highest IFNγ expression upon being co-cultured with the primary OC cells.

### 2.6. Dual-CAR

**Figure 5:**
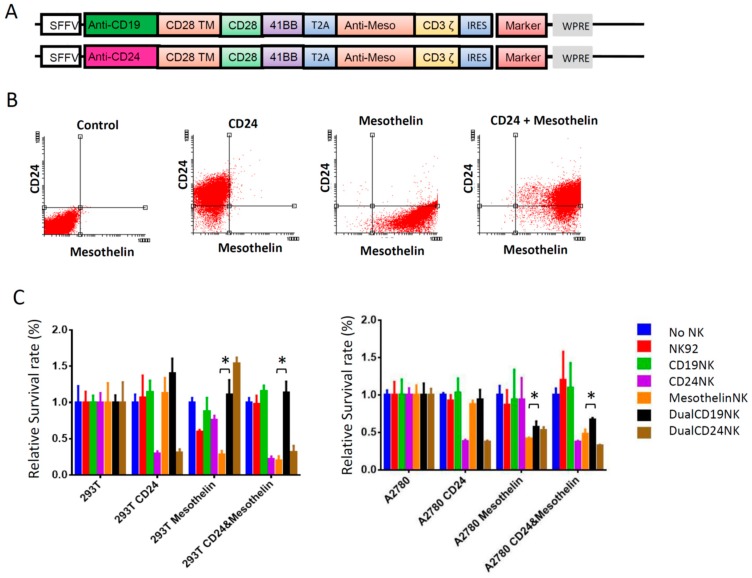
Anti-CD24/anti-mesothelin dual-CAR-NK-92 cells show killing activity against cancer cells expressing both antigens. (**A**) Schematic illustration of the structure of the dual-CAR based on the concept of Wilkie et al. [18]. The co-stimulatory domains are split over both CARs. The anti-CD24 scFv is fused to the CD28 and 4-1BB domains. The anti-mesothelin scFv is fused to the CD3ζ signaling domain. The two CARs are connected via a T2A sequence to allow equal expression of both from a single promoter. (**B**) FACS analysis of HEK293T cells which were previously transduced with the respective antigens. (**C**) Results of a FluoroSkan-based assay. CD24, mesothelin or CD24 and mesothelin expressing HEK293T and A2780 cells were co-incubated with the different NK-92 cells targeted against the indicated antigens at an E/T ratio of 5:1 over 24 h. * indicate *p* < 0.005 (unpaired *t*-test). Values represent the mean from three different samples of two separate experiments.

As demonstrated above, the new anti-CD24–CAR-NK-92 cells showed very high cytotoxic activity against antigen-presenting cells, but also limited activity against CD24-negative cells. With the goal to prevent possible off-target effects in our model system and the respective clinical trials, we aimed to restrict the cytotoxic effect to cells presenting two antigens using a dual-CAR-approach [18]. Mesothelin was selected as a co-antigen because mesothelin is broadly expressed in OC cells and its role as an immunotherapeutic antigen has been evaluated in previous studies [15,19]. Also, mesothelin was frequently expressed in our available OC cell lines: OVCAR3 (73%) and SKOV3 (25%). All primary OC cells also showed high mesothelin expression levels (39%–67%). Only A2780 cells were negative for mesothelin expression. Figure 5A illustrates the dual-CAR design tested here, which consisted of an anti-Mesothelin-CD3ζ CAR connected via a T2A esterase site to an anti-CD19- or anti-CD24 scFv fused to CD28 and 4-1BB co-stimulatory domains. Figure 5C presents the results of the corresponding cytotoxicity experiment. We seeded HEK293T and A2780 cells previously equipped with CD24, mesothelin or both antigens and co-incubated these target cells with unmodified NK-92 cells, or NK-92 cells engineered to express the indicated mono- and dual-CARs. Fluoroskan was performed to assess antigen-presenting cells after co-incubation with the various NK-92 cell populations for 24 h at an E/T ratio of 5:1. As expected, there was a significantly reduced killing effect of the anti-CD24-dual-CAR compared to the third-generation anti-mesothelin-CAR when co-cultured with solely mesothelin positive target cells (*p* < 0.005). However, this effect was not observed regarding CD24, where the dual-CAR-NK-92 cells showed a similar activity as that observed for the CD24-CAR-NK-92 cells against target cells expressing only CD24 (HEK293T *p* = 0.567, A2780 *p* = 0.594) (Figure 5C).

## 3. Discussion

In this study, we present a new third-generation CAR against CD24, a promising antigen overexpressed in multiple tumors with high prevalence on CSCs [6], and analyzed its functionality in CAR-NK cells in OC cell lines and patient-derived primary tumor samples. We showed that CAR-engineered NK-92 cells exhibited specific and strong cytotoxic activity against antigen-presenting OC cell lines and, importantly, primary OC cells. A dual-CAR approach allowed for a combinatorial treatment against a CSC-specific antigen (CD24) and a common OC antigen (mesothelin).

Adoptive immunotherapy with CAR effector cells represents one of the most promising anti-tumor strategies at the moment. In several diseases, CAR-engineered T-cells are tested against different antigens that display varying levels of tumor specificity [15,16,17,20]. In OC, CAR-based immunotherapy is also currently exploited by several groups [15,16,20]. However, the first clinical study failed to show a clinical benefit of anti-FRα-CAR-based immunotherapy with T-cells [20]. Since then, several optimizations for CAR design, target selection and study design have been proposed [21]. Similar to antibody-based treatments, immunotherapy with CAR effector cells against one specific antigen will not suit all patients. According to this, multiple CARs against several antigens will have to be developed and tested.

Although medical progress advances exponentially in several diseases, primary OC treatment is still based on extensive abdominal surgery and platin-based adjuvant chemotherapy [1,22]. Despite macroscopic tumor removal, OC recurs peritoneally in most cases and ultimately leads to death. The explanation for this might be found in the model of CSCs, a subpopulation of highly tumorigenic cancer cells with an increased potential for chemoresistance and metastatic spread [19,23,24,25,26]. CAR-based immunotherapy directed against antigens that characterize CSC or their abilities, represents a very promising therapeutic approach. Several CSC-associated antigens were described and the first CAR studies targeting these antigens were performed [27,28].

In this study, we chose CD24 as a promising target as anti-CD24 immunotherapy has already been tested in other malignancies and showed promising results [9,10,11]. Previous studies have also demonstrated that CD24 is a marker for CSCs [6,7,10].

CD24 is a P-selectin ligand, which is normally found on activated platelets and endothelial cells. The binding of CD24 to P-selectin facilitates tumor cell survival and dissemination [29,30]. Additionally, CD24-positive OC cells showed an EMT phenotype with higher invasive growth compared to non-expressing tumor cells as described for CSCs [8,31]. Interestingly, anti-CD24 treatment with the monoclonal antibody SWA11 strongly changed the intratumoral cytokine microenvironment in mice [11]. These findings indicate that CD24 is a promising target in OC immunotherapy. Targeting CD24 has been shown to induce tumor cell apoptosis and effectively suppress hepatocellular carcinoma in mice bearing HCC xenografts [9]. In addition, CAR-CD24 T cells inhibited growth and metastasis of pancreatic adenocarcinoma xenografts in mice [10].

In contrast to most other studies using CAR therapy, we chose NK cells over T-cells [15,16,17,20,32]. In addition to CAR-mediated killing, NK cells also exhibit tumor-associated antigen unrestricted killing via the expression of several natural cytotoxicity receptors. This enables NK cells to also lyse antigen-negative tumor cells [32]. NK-92 cells, a cell line derived from a patient with lymphoma, were tested in several preclinical and clinical studies with only moderate and transient toxicities [33,34]. This cell line expresses many activating receptors and cytolytic pathway molecules, like perforin and granzyme B [35]. NK-92 cells can easily be cultured, genetically modified and expanded. In contrast to autologous cells, NK-92 cells can quickly be provided in the desired amount and quality. NK-92 cell infusions were well-tolerated and no long-term side effects were observed, thus NK-92 cells appear to be a promising cell line for adoptive cancer therapy [33,34].

A previous clinical study using a first generation anti-FRα CAR in a T-cell-based immunotherapy was unable to demonstrate a clinical benefit in OC patients, although preclinical studies showed strong killing activity of CAR-engineered T-cells against tumor cells [20]. The clinical failure of many first generation CAR-T cell therapies was thought to be due to the lack of costimulatory domains. That is why we selected a third-generation CAR backbone that additionally expresses CD28 and 4-1BB as costimulatory domains. These domains enhance the proliferation and survival of T-cells [13,36].

NK-92 cells equipped with the newly designed anti-CD24 CAR showed very high cytotoxicity against CD24-expressing OC cell lines and primary cells. The cytotoxic effect depended on antigen expression level and sensitivity of target cells to NK cell-mediated killing. As shown in our experiments, SKOV3 cells appeared to be more resistant to unspecific killing by control cells compared to A2780 cells, which expressed lower levels of CD24. In primary OC cells, the higher CD24-expressing P2 cells were more readily killed by control NK-92 cells (P2 and P3 vs. P1). In parallel, the measurement of NK cell activation by IFNγ secretion assay confirmed these observations. This fact has to be kept in mind when transferring positive results from one cell type to another, or even a different stage of disease, in order to avoid overestimation of CAR-restricted killing effects. However, this probably shows the potential of NK-92 cells to also kill tumor cells in an antigen-unrestricted manner.

Compared to other CARs we tested in our lab, the anti-CD24 CAR was superior in terms of cytotoxic effect and killing dynamics, even against target cells with low antigen expression (data not shown). This might be explained by the high affinity and the binding site of the implemented scFv [12,37]. Experiments to evaluate whether the killing was restricted to CD24 positive cells showed that only antigen-expressing cells were specifically targeted and significantly killed (Figure 2). Additionally, we were able to directly observe fast killing dynamics and proliferation of engineered NK cells. This might be of particular importance in solid tumors. The greatest obstacle in cell-based immunotherapy for solid tumors is the tumor microenvironment, which influences the immune system with multiple pro- and anti-inflammatory signals [38,39]. In contrast to the successful results in clinical studies for hematological diseases, CAR therapy failed in several clinical T-cell-based studies for solid tumors, as previously described for OC [20,40]. The less-vascularized and hypoxic tumor microenvironment hinders effector cells to infiltrate the tumor and expand. Additionally, quiescent CSCs, which probably do not express the targeted tumor-specific antigen, will survive and lead to disease recurrence. Several strategies are being developed to try to overcome these limitations, such as antigen selection, preconditioning, CAR affinity modifications, or T-cell selection [41]. A very promising approach which we plan to implement in further studies is to influence the tumor microenvironment by locally produced cytokines, as recently shown for the TRUCK concept [27]. Additionally, switching from T-cells to different effector cells might be a promising strategy. NK cells might be advantageous over T-cells regarding this aspect due to their native anti-tumor activity and their specific immune-modifying cytokines [42]. Upon antigen presentation, we observed high and specific activation of NK cells as indicated by IFNγ secretion. This effect was especially prominent upon NK-92 co-culture with primary OC cells, with much higher IFNγ secretion in co-cultures containing CD24-CAR-NK-92 cells. However, even the co-culture of CD24-CAR-NK-92 cells with low CD24-expressing target cells like A2780 or primary OC sample P1 led to elevated IFNγ expression. This observation might be explained by the antigen-unrestricted anti-tumor activity of NK cells as described above. Importantly, control CD19-CAR-NK-92 cells did not cause more killing activity against CD24-expressing target cells than untransduced control NK-92 cells. A library of universally applicable, short-lived NK-92 cells represents a promising therapeutic strategy against several solid tumors. We already know from previous studies that in vivo persistence of CAR-T-cells appears to be necessary for long-lasting therapeutic effects. On the other hand, short-lived cells might lead to a better control of toxicities. Repetitive infusions of shortly persisting CAR-NK cells targeting variable antigens (common tumor associated antigens, CSC antigens) in combination with already established adjuvant therapies or additive long-lasting immunotherapies could be a successful strategy. Therefore, NK-92 cells seem to be an ideal candidate [42].

However, further studies evaluating the effect of NK cell-based adoptive immune therapy in solid tumors are urgently needed to support these observations and ideas.

A major concern in adoptive immune therapy is the risk for off-target effects [40]. CD24 is not only found in tumor cells, but is also expressed in hematologic cells (monocytes, granulocytes, B/T lymphocytes) at variable levels [6]. Highly cytotoxic third-generation CARs potentially harbor a greater risk for severe off-target effects. On the other hand, first-generation CARs did not exhibit significant killing effects in solid tumors in clinical trials [20,40]. There are several options discussed to prevent severe off-target effects [21]. Primary NK cells have a significantly shorter lifetime compared to T-cells [32]. Their clinical effect can, thereby, be better controlled and observed. Another option is to find a highly specific antigen, which is only expressed in cancer cells.

Another strategy is to create a more specific engineered effector cell by combining two CARs against different antigens in a costimulatory design, while splitting the costimulatory domains over both CARs as described by Wilkie et al. [18]. This led us to combine our new CAR against the potential CSC marker CD24 with a newly designed CAR against the common OC antigen mesothelin. In contrast to the earlier publication, we included the costimulatory domain 4-1BB in addition to CD28 in our dual-CAR.

Interestingly, we only observed higher specificity of the dual-CAR compared to the third-generation single-CAR against mesothelin-positive cells, but not against CD24-positive cells. In contrast to some studies using dual-CAR strategies to increase effector cell specificity, we did not observe killing to be restricted solely to double-positive target cells [43]. Earlier studies investigating this approach also saw activity against single-positive cells [18,41]. However, this was only observed against the antigen that was targeted by the first-generation CAR. Interestingly, in our NK cell-based design, we also saw killing activity against the target by the incomplete CAR, which lacked the CD3ζ domain. A possible explanation might be the high affinity of our CD24 CAR and a different function of this dual-CAR approach in NK cells as compared to T-cells. Until now, this approach had only been tested in T-cells. Due to the antigen-unrestricted killing activity of NK cells, a transient binding to the antigen presenting cell might induce killing even without activation of the complete intracellular costimulatory signaling that should occur upon binding of both CARs. However, we constructed a highly efficient single-vector CAR design against two different antigens in NK cells that might facilitate a multi-target approach for OC. Further studies are required to understand the exact mechanism behind this persistent killing activity and the functionality of this approach in NK cells. Nevertheless, one major disadvantage of this system will always be the background activity mediated by the first-generation CAR. Therefore, we will investigate further strategies in dual-CAR designs. For example, others described another dual-CAR design using a sequential activation over a synthetic Notch receptor, which is an option for further experiments [44].

In vivo studies are needed to verify the function of the new CAR and evaluate potential off-target effects. Furthermore, we are planning to analyze the effect of this CAR in combination with chemotherapy, a strategy which was shown to be very promising in earlier studies [19,45].

In summary, we developed a new anti-CD24 CAR against ovarian cancer. Our new anti-CD24 third-generation CAR proved to be highly specific with strong cytotoxic activity against CD24-positive OC cell lines and primary cells. An NK-92 based approach proved to be feasible and effective in our in vitro experiments. A dual-CAR approach with split costimulatory domains might be useful to create a more specific and efficient effector cell against two separate antigens. This data represents the basis for further in vivo studies and future progress to clinical trials.

## 4. Material and Methods

### 4.1. Cell lines

HEK-293T cells (ATCC CRL-3216) were used for lentiviral vector production (see below). In cytotoxicity experiments, we used the already established human ovarian cancer cell lines SKOV3, A2780, OVCAR3 and the human NK cell line NK-92 [42]. After obtaining informed consent, primary ovarian cancer cells were harvested from sequential ascites samples of an OC patient and cultured in low-attachment flasks (Corning, Wiesbaden, Germany). This study was approved by the local ethics committee (nr. 179/14-ff, approved 26 June 2014). OC cell lines and HEK-293T cells were equipped with GFP by lentiviral transduction. HEK-293T cells were cultured in DMEM (Biochrom, Berlin, Germany) supplemented with 10% heat inactivated FBS, 100 U/mL penicillin, 100 mg/mL streptomycin, and 10 mmol/L HEPES. Ovarian cancer cell lines were cultured similarly but in RPMI-1640 (PAN Biotech, Aidenbach, Germany) instead of DMEM.

### 4.2. Cloning of Vectors and Virus Production

The sequence of the scFv was codon-optimized for human codon usage; cryptic splice sites were removed and the GC content was improved to optimize transcriptional processing and protein expression. The sequence was synthesized by GeneART (Thermo Fisher, Regensburg, Germany). The scFv was cloned into a third-generation CAR containing CD28, CD137 (4-1BB) and CD3ζ domains. The sequence was adapted from an already published third-generation anti-CD19 CAR, which served as a control in further experiments [46,47]. To generate a 3^rd^ generation lentiviral SIN vector driven by an internal SFFV U3 promoter [48], a three-fragment ligation (using *Not*I, *Sal*I and *Age*I restriction sites) was performed to fuse the scFv, CD28-41BB-CD3ζ and lentiviral backbone fragments. An IRES (internal ribosomal entry site)-driven dTomato expression cassette was inserted between *Sal*I sites to allow co-expression and facilitate detection.

All plasmids for lentiviral packaging were produced and purified at the Plasmid Factory (Bielefeld, Germany). Transfection was performed in HEK-293T cells (5 × 10^6^, 10 cm Dish). The following plasmids were used in a calcium phosphate-based transfection: 12 μg of the lentiviral vector plasmid, 12 μg of pcDNA3.GP.4×CTE (gag/pol), 5 μg of pRSV-Rev, and 2 μg of RD114/TR envelope plasmids [48]. Lentiviral supernatants were harvested 36 h after transfection, filtered through Millex-GP 0.22 μm filters (Millipore, Schwalbach, Germany), concentrated via ultracentrifugation, and stored at −80 °C.

### 4.3. Transduction of NK Cells

A retronectin-assisted transduction was conducted to equip NK-92 cells with the designated CARs according to the following protocol. After coating 48-well plates with Retronectin (Takara, Shiga, Japan, 210 µL of 24 mg/mL in PBS per well) overnight, the wells were blocked with sterile-filtered PBS containing 2% BSA for 30 min followed by washing with HBSS/HEPES. Then, lentiviral supernatants were added and the plates were centrifuged for 30 min at 400× *g* at 4 °C. Afterwards, 5 × 10^4^ NK-92 cells were added and incubated for 24 h.

### 4.4. Antibodies and ELISA

The Human IFN-gamma DuoSet ELISA (R&D Systems, Minneapolis, MN, USA) was used to measure IFNγ concentrations in the supernatants according to the manufacturer’s instructions. Anti-CD24 antibodies were purchased from Miltenyi Biotec (Miltenyi Biotec GmbH, Bergisch Gladbach, Germany).

### 4.5. Cytotoxicity Assays

#### 4.5.1. Fluoroskan Ascent™ FL

First, we seeded OC cells in flat bottom 96-well plates (Sarstedt, Nürmbrecht, Germany) at appropriate densities, which were determined earlier (A2780, 2 × 10^4^ cells/well; SKOV3 and OVCAR3 1.5 × 10^4^ cells/well). The following day, NK-92 cells were added at the designated effector/target (E/T) ratios. At several time points, fluorescence was measured using the microplate fluorometer Fluoroskan Ascent™ FL (Thermo Fisher Scientific, Waltham, MA, USA). Prior to acquiring fluorescence measurements to quantify OC cells, the culture medium was completely removed by inverting the plates and blotting them against clean paper towels. This served to remove the NK-92 suspension cells from the wells. Two-hundred microliters 5% (*w*/*v*) SDS was added into each well, followed by detection of the fluorescence intensities of GFP in the cell homogenate with excitation at 485 nm and emission at 520 nm.

#### 4.5.2. xCELLigence

For real-time analysis of NK-specific killing of unmarked target cells, we used the xCELLigence RTCA SP instrument (ACEA Biosciences, San Diego, CA, USA). Before seeding the target cells, 100 µL of cell culture media was added to each of the 96 wells in the E-Plate 96 (ACEA Biosciences). This equilibration step was performed for 30 min at room temperature. Next, the background impedance of cell culture media was measured as the reference impedance, which is necessary to calculate the cell index (CI) value. Afterwards, the E-Plate 96 was removed from the incubator and the desired cells were added in 50 µL medium. The appropriate cell concentrations were previously determined by titration experiments (A2780: 2 × 10^4^; OVCAR3: 1.5 × 10^4^; SKOV3 and primary ovarian cancer cells: 1 × 10^4^). The plate was left in the culture hood for 30 min to allow the cells to slightly attach. Then the E-Plate 96 was reinserted. The next day, when the cells reached the logarithmic growth phase, effector NK cells were added in 50 µL medium at the desired E/T (effector to target) ratio. The experiments were manually stopped 48 to 72 h after the addition of effector cells.

## Supplementary Materials

Supplementary materials can be found at http://www.mdpi.com/1422-0067/20/3/660/s1. Supplement video 1 (E/T 1:1) and 2 (E/T 2:1) of a co-culture of anti-CD24-CAR-NK92 cells with CD24 positive OVCAR3 cells (green) and CD24 negative HEK-293T cells (grey) under the fluorescence microscope. Pictures were taken every 10 min.
